# Is there an indication for surgery in patients with spinal deformities? – A critical appraisal

**DOI:** 10.4102/sajp.v77i2.1569

**Published:** 2021-10-04

**Authors:** Hans-Rudolf Weiss, Xiaofeng Nan, Matthew A. Potts

**Affiliations:** 1 Schroth Best Practice Academy, Neu-Bamberg, Germany; 2 Nan Xiaofeng’s Spinal Orthopedic Workshop, Xi ‘an, China; 3 Dorsi Spinal Institute, Nottingham, United Kingdom

**Keywords:** spinal deformities, scoliosis, kyphosis, spine surgery, evidence, indications

## Abstract

**Background:**

High-quality evidence exists to support physiotherapy and brace treatment for scoliosis and other spinal deformities. However, according to previous systematic reviews, it seems that no evidence exists for surgery. Nevertheless, the number of research articles focussing on spinal surgery highly exceeds the number of articles focussing on conservative treatment.

**Objective:**

The purpose of this study is to conduct an updated search for systematic reviews providing high-quality evidence for spinal surgery in patients with spinal deformities.

**Method:**

A narrative review including PubMed and the Cochrane database was conducted on April 12, 2020, with the following search terms: (1) spinal deformities, surgery, systematic review and outcome; (2) kyphosis, surgery, systematic review and outcome; (3) Scheuermann’s disease, surgery, systematic review and outcome, and (4) scoliosis, surgery, systematic review and outcome.

**Results:**

No reviews containing prospective controlled or randomised controlled studies were found providing evidence for surgery.

**Conclusions:**

A general indication for spine surgery just based on the Cobb angle is not given. In view of the long-term unknown variables and the possible long-term complications of such treatment, a surgical indication for patients with spinal deformities must be reviewed on an individual basis and considered carefully. A current systematic review appears necessary in order to be able to draw final conclusions on the indication for surgery in patients with spinal deformities.

**Clinical implications:**

In view of the increasing number of surgeons with an affiliation to industry, the indication for surgery needs to be given by independent conservative specialists for spinal deformities in order to provide an objective recommendation.

## Introduction

Spinal deformities can be sub-divided into sagittal plane deformities (e.g. kyphosis, lordosis) and frontal plane deformities such as scoliosis (Chik 2020). Whilst kyphosis can appear as a sole sagittal disorder of the spine, it can also appear in combination with a scoliosis (Turnbull & Weiss 2020). Kyphosis may be of adolescent onset (Turnbull & Weiss 2020), for example, Scheuermann’s disease, or appear in the elderly population as degenerative kyphosis (Ng 2020). More rarely, there may be a congenital kyphosis comprising the sagittal plane only (Kaspiris, Weiss &, Moramarco 2020).

In scoliosis patients, the frontal plane deformity might be the most obvious; however, scoliosis must be regarded as a three-dimensional deformity with a frontal deviation, with trunk rotation or torsion and with a sagittal plane alteration (Asher & Burton 2006; Goldberg et al. 2002; Kruzel & Moramarco 2020; Lonstein 1995). Scoliosis may develop because of neuromuscular alterations, mesenchymal disorders, congenital malformations and other rare diseases (Chik 2020). The majority of scoliosis cases are idiopathic when an underlying cause cannot be identified. Idiopathic scoliosis can be sub-classified as early-onset idiopathic (infantile and juvenile type) or late-onset idiopathic scoliosis with an onset at 10–14 years of age in adolescent idiopathic scoliosis (AIS) (Kruzel & Moramarco 2020).

**FIGURE 1:**
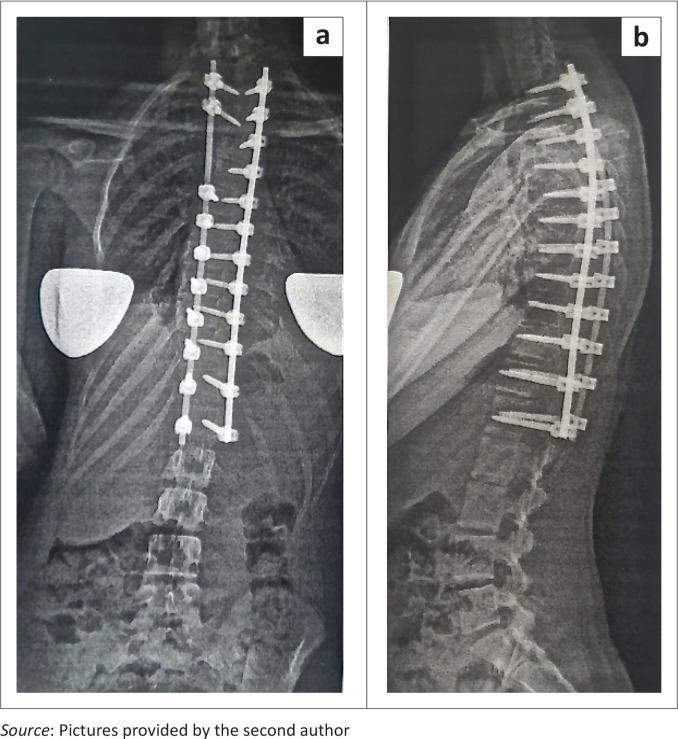
(a) Dorsal double rod instrumentation with (b) pedicle screws for a patient with adolescent idiopathic scoliosis.

**FIGURE 2:**
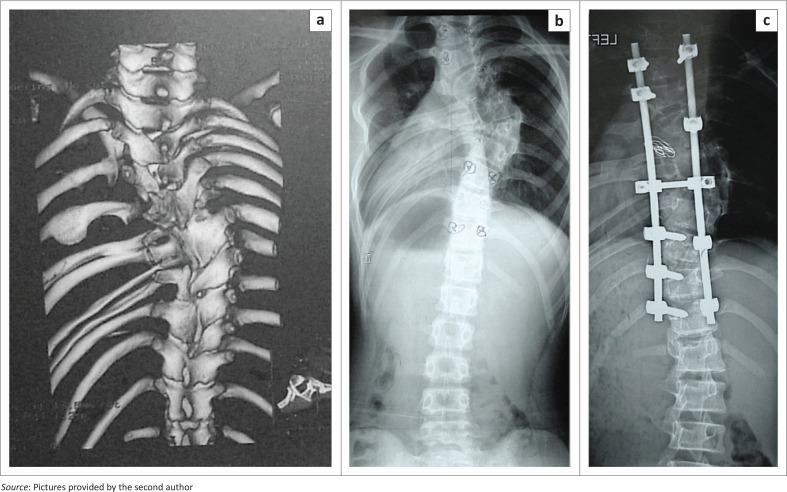
(a) CT reconstruction of the spine and ribs in a patient with congenital scoliosis including vertebral malformations and a segmentation disorder (rib synostosis). *(b)* Native x-ray, and (c) Spine after dorsal double rod instrumentation with pedicle screw fixation.

In 80% – 90% of all scoliosis cases, AIS is the most frequent type (Asher & Burton 2006; Goldberg et al. 2002; Kruzel & Moramarco 2020; Lonstein 1995). The prognosis of a Scheuermann’s kyphosis and AIS in principle is benign (Asher & Burton 2006; Goldberg et al. 2002; Kruzel & Moramarco 2020; Lonstein 1995; Turnbull & Weiss 2020). Life-threatening issues are rare, even if the condition stays untreated. In late adulthood, untreated patients with AIS function well and have no other complaints other than (non-disabling) low back pain and cosmetic concerns (Asher & Burton 2006; Weinstein et al. 2003; Weiss et al. 2016b). Early-onset idiopathic scoliosis and other paediatric deformities, however, if untreated, may progress to 100° or more after growth-causing restrictive ventilation disorders and cor pulmonale in adulthood (Pehrsson et al. 1992; Weiss & Turnbull 2020). Patients with significant curvatures have a lower life expectancy (Cunin 2015; Pehrsson et al. 1992; Weiss & Turnbull 2020). The treatment of spinal deformities consists of physiotherapy (alone in mild curves), brace treatment and spinal surgery. High-quality evidence exists to support physiotherapy and brace treatment (Kuru et al. 2016; Monticone et al. 2014; Nachemson & Peterson 1995; Weinstein et al. 2013; Weiss & Weiss 2005), but according to some previous reviews, there is no evidence for surgery (Figures 1 and 2) in patients with a spinal deformity (Bettany-Saltikov et al. 2015; Cheuk et al. 2015). Nevertheless, the number of articles focussing on spinal surgery highly exceeds the number of articles focussing on conservative treatment (Hawes 2006).

The indication for surgery in patients with a spinal deformity mainly is based on the angle of curvature (Cobb 1948). Whilst for patients with AIS, the indication is usually set at an angle of 50° (Asher & Burton 2006; Kruzel & Moramarco 2020; Weiss 2008), the indication is set at a sagittal Cobb angle of 70° for patients with a thoracic kyphosis (Bradford et al. 1975; Cobden et al. 2019; Polly et al. 2019). The indication for surgery on patients with spinal deformities in adolescence must therefore be viewed as preventive therapy, carried out at an age at which no symptoms have yet occurred. Such an intervention is intended to prevent functional disorders in the musculoskeletal and cardiorespiratory areas.

The purpose of this study was to search for systematic reviews providing high-quality evidence for spinal surgery in patients with spinal deformities in order to support the indications currently applied.

## Materials and methods

A narrative review including PubMed and the Cochrane database was conducted on 12 April 2020, with the following search strings: (1) spinal deformities, surgery, systematic review, outcome; (2) kyphosis, surgery, systematic review, outcome; (3) Scheuermann’s disease, surgery, systematic review, outcome and (4) scoliosis, surgery, systematic review, outcome. Reviews have been extracted containing information about the evidence for and complications of surgery for patients with scoliosis and/or kyphosis.

These four search strings served as the basis for the detection of studies providing high-quality evidence (prospective controlled or randomised controlled studies) in order to support surgical treatment of patients with spinal deformities. Reviews comparing surgical techniques to each other, reviews on fractures, spondylolisthesis, osteoporosis and disc degeneration as well as on spinal stenosis were excluded.

The search was carried out by the first author according to the following inclusion criteria:

1. systematic review
2. scoliosis and/or kyphosis
3. spinal surgery (for correction of a spinal deformity)
4. outcome mentioned in the abstract, and
5. complications of surgery.

All five inclusion criteria were accepted as well as 1–3 and 4 or 1–3 and 5.

Exclusion criteria were:

1. abstract out of topic, and
2. one of the inclusion criteria 1–3 missing.

***Appraisal tool*:** CASP checklist (Critical Appraisal Skills Programme 2018), a qualitative checklist without a score.

### Ethical considerations

All patients and their parents have agreed to the publication of their pictures and x-rays within scientific articles.

## Results

Because only a few systematic reviews were found comprehensive narrative reviews (Hawes 2006; Hawes & O’Brien 2008; Weiss & Goodall 2009) were accepted. For search (1), 257 items were found. Ten (10) of these articles are relevant with respect to the search string (Bettany-Saltikov et al. 2015, 2016; Hawes & O’Brien 2008; Lau et al. 2014; Sharma et al. 2013; Weiss 2008; Weiss & Goodall 2008a, 2009; Yadla et al. 2010; Zanirato et al. 2018).

For seach (2), 57 items were found, and nine were identified as relevant (Bettany-Saltikov et al. 2015, 2016; Cho, Shin & Kim 2014; Fu et al. 2016; Guan, Zhang & Xu 2020; Huq et al. 2019; Kim et al. 2012; Lau et al. 2014; Tsirikos 2009).

For search (3), three items were found, and two of them were identified as relevant (Huq et al. 2019; Tsirikos 2009).

For seach (4), 116 items were found, and 26 items were identified as relevant (Aghdasi et al. 2020; Bettany-Saltikov et al. 2015, 2016; Cho et al. 2014; Drazin et al. 2011; Evaniew et al. 2015; Figueiredo et al. 2016; Guan et al. 2020; Hawes & O’Brien 2008; Kim et al. 2012; Larson et al. 2013; Lau et al. 2014; Ledonio, Polly & Crawford 2013; Legg et al. 2014; Liang et al. 2012; Sharma et al. 2013; Theis, Gerdhem & Abbott 2015; Toovey et al. 2017; Weiss 2008; Weiss & Goodall 2008a, 2009; Wu et al. 2019; Yadla et al. 2010; Yang et al. 2016; Yoshihara 2019; Zanirato et al. 2018).

Eleven studies appeared in more than one search (Bettany-Saltikov et al. 2016; Cho et al. 2014; Guan et al. 2020; Hawes & O’Brien 2008; Kim et al. 2012; Lau et al. 2014; Sharma 2013; Weiss 2008; Weiss & Goodall 2008a, 2009; Yadla et al. 2010).

No reviews containing prospective controlled or randomised controlled outcome studies were found within the investigated reviews.

Few systematic reviews (Liang et al. 2012; Sharma 2013; Weiss & Goodall 2008b; Yadla et al. 2010; Zanirato et al. 2018) were found with respect to complications with one exception. The article by Weiss and Goodall (2008b) was published as a systematic review, but it does not meet the criteria of a systematic review as it is required today. Also, non-systematic reviews (Hawes 2006; Hawes & O’Brien 2008; Weiss, Moramarco & Moramarco 2013) have been taken into account for this search with a focus on articles including follow-ups exceeding 2 years. The literature of these narrative reviews was also searched for long-term results.

Complications that were reported in the relevant articles were, for example, severe blood loss, urinary infections because of catheterisation, pancreatitis and obstructive bowel dysfunction as a result of immobilisation during and after surgery, early infection and inflammatory processes, post-surgery chronic pain, failure of the instrumentation, misplacement of instrumentation, decompensation and increased sagittal deformity, increased torso deformity, late infections (sometimes with onset more than five years after surgery), the need for salvage surgery, death and neurological damage. Hicks et al. (2010) have found that malposition is the most commonly reported complication of thoracic pedicle screw placement, at a rate of 15.7% per screw.

High rates of early complications have been found mainly in surgery applied for paediatric deformities as well as for adult deformities (Cho et al. 2014; Drazin et al. 2011; Figueiredo et al. 2016; Fu at al. 2016; Guan et al. 2020; Hawes & O’Brien 2008; Kim et al. 2012; Ledonio et al. 2013; Legg et al. 2014; Sharma 2013; Toovey et al. 2017; Wu et al. 2019:682–693; Yadla et al. 2010; Zanirato et al. 2018). In the systematic review by Zanirato et al. (2018), the rate of complications for patients with adult spine deformity (ASD) is dependent on the surgical technique applied, between 24% and 36% perioperative plus 11% and 15% late complications.

The rate of complications varies widely within the articles found. Whilst in AIS cohorts the complication rate within the first 2 years seems rather low (Weiss & Goodall 2008b) in a study with a follow-up time of 5–20 years, the rate of reoperations was 48% with half of the population operated for late infections, the other half for chronic pain appearing after surgery (Mueller & Gluch 2012).

## Discussion

The indication for treatment results from a proven effect and an adequate risk assessment. If an effect has not been proven or if the risks outweigh the beneficial effects, treatment is not indicated. An indication for treatment may only be made if the risk/reward ratio is favourable for the patient.

Although the overall number of publications on spinal surgery in patients with spinal deformities is high (Hawes 2006), no conclusive evidence has been found in our narrative review to support this procedure. Neither general systematic reviews nor Cochrane reviews have been found within our narrative review to support spinal surgery for spinal deformities (Bettany-Saltikov et al. 2016; Cheuk et al. 2015; Hawes 2006; Hawes & O’Brien 2008; Ward et al. 2017; Weiss 2008; Weiss & Goodall 2008b, 2009). Therefore, we may conclude that there is no general indication just based on the Cobb angle (the most important measurement for measuring the severity of a spinal curve) for spinal surgery in patients with spinal deformities (Hawes 2006:318–339; Moramarco 2013; Weiss & Goodall 2008b; Weiss et al. 2013) and the lack of evidence as found in our search. In contrast, the Iowa studies have shown that AIS patients function well in older age. The last study of the study series from Iowa had a 50 year follow-up of untreated cases with late idiopathic scoliosis (LIS) with most of the patients at an age of more than 65 years (Weinstein et al. 2003). A more recent review supports this point of view (Weiss et al. 2016a). Therefore, preventive surgery in patients with spinal deformities must be viewed as an optional surgery. Accordingly, it must not be indicated by the spinal surgeon but must be selected by the patient.

**FIGURE 3:**
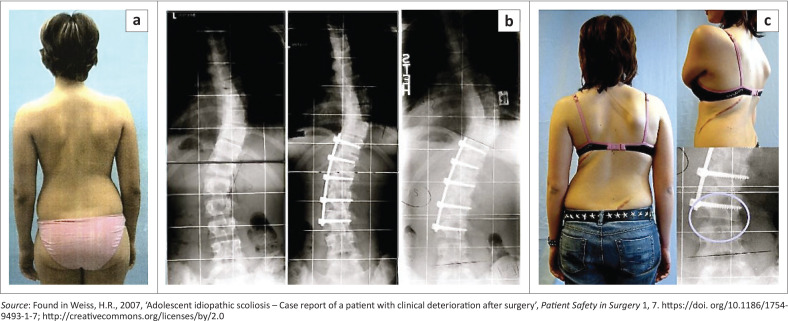
(a) 14-year-old, almost fully mature girl with AIS and balanced appearance, (b) native X-ray before and after the operation. (c) Appearance after surgery with clear decompensation and radiologically visible wedge deformation of the intervertebral space below the spinal fusion.

However, as pointed out below (Beschloss et al. 2021), the number of surgeries for patients with spinal deformities is constantly rising, and this fact is consistent with the experience of the first author. The question therefore arises why today there are still so many surgeries performed mainly in otherwise healthy individuals with a benign condition like AIS (80% – 90% of all scoliosis cases), sometimes even in curves of less than 40°, and in relatively mature patients without a significant risk for further deterioration (Weiss 2007, see Figure 3). Scheuermann’s kyphosis is a relatively benign disorder as well with a certain risk of chronic low back pain (Grauers et al. 2014) especially in cases with a lumbar kyphosis (Weiss & Turnbull 2019).

To answer this question, the following subject areas are to be outlined that spinal surgeons regularly use when they want to make the need for spinal surgery plausible to those affected, and their relatives.

### Back pain and spinal deformities

Actually, there is no evidence that spine surgery would prevent low back pain in the long term (Danielsson & Nachemson 2003; Upasani et al. 2008; Weiss et al. 2016b). The best predictor for having back pain after surgery obviously is back pain before surgery (Hwang, Pendleton & Samdani 2020). Post-surgical patients might have about the same amount of back pain as untreated patients with spinal deformities (Danielsson & Nachemson 2003), sometimes also disabling pain leading to revision surgery (Mueller & Gluch 2012; Zhang & Zhang 2020). There is some evidence that pain may increase over time after surgery (Upasani et al. 2008). The argument that pain statistically may increase with increasing Cobb angles and that health-related quality of life (HRQoL) may decrease is basically true (Danielsson & Nachemson 2003). Nonetheless, should this lead to a surgical indication in an otherwise healthy 14-year-old girl? Most patients with AIS or a Scheuermann’s kyphosis treated conservatively do not suffer from chronic pain during adolescence or during their early adulthood. Obviously, pain is a more common symptom exhibited by older adults; however, according to a 50-year follow-up of patients with untreated idiopathic scoliosis, this pain is not disabling (Weinstein et al. 2003) and may successfully be treated conservatively when it appears in the individual case (Zapata et al. 2015).

### Spine surgery and torso deformity

**FIGURE 4:**
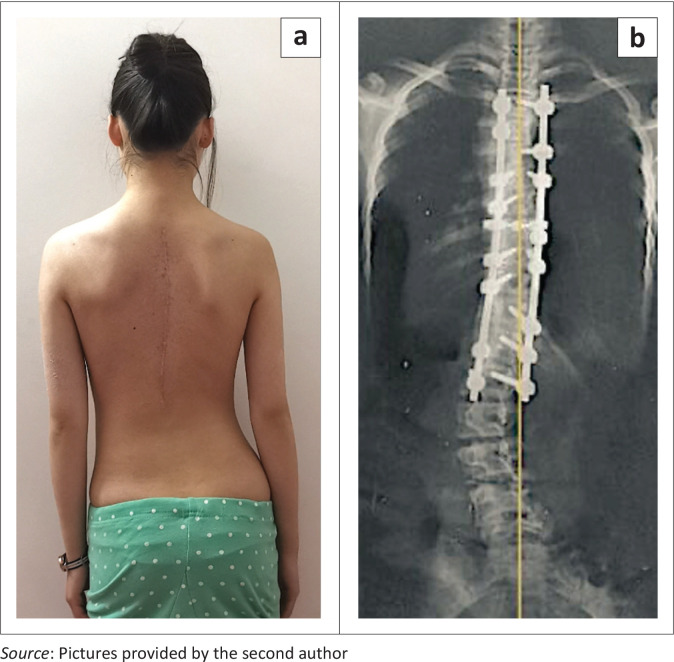
(a) Dorsal double rod instrumentation with full correction of a main thoracic curve initially exceeding 50°. (b) The unfused short lumbar counter-curve to the left post-surgery leads to a decompensated posture with pelvic prominence on the right.

**FIGURE 5:**
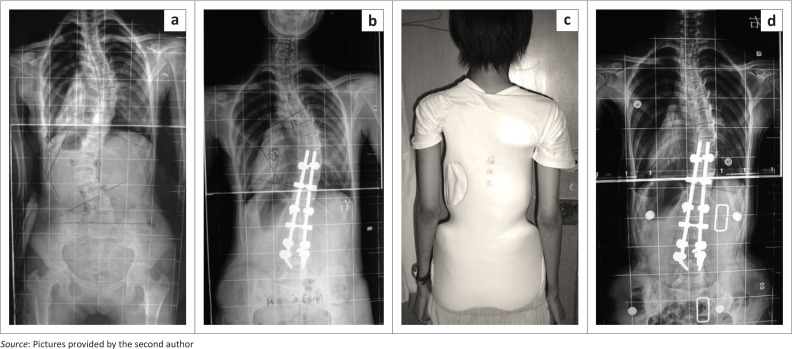
(a) X-ray of a patient with a Lenke 3C pattern before undergoing surgery. (b) Only the lumbar part was fused, and because of the decompensation of the thoracic curve, a brace (c) was made with moderate correction of the thoracic curve (d). With appropriate planning of this surgery, these issues would have been avoidable.

As a matter of fact, the impact of spinal surgery on the torso deformity is limited. It is well-known that after surgery, the rib hump may reappear within a year (Cui et al. 2012; Hawes 2006; Hawes & O’Brien 2008; Lau et al. 2014; Weiss 2007; Weiss & Goodall 2008a). Considering this, even cosmetic reasons are not an indication for surgery. However, surgery in some cases may even lead to a deterioration of the trunk deformity (Weiss 2007 and Figure 3, Figure 4 and Figure 5).

### The matter of progression in adult age

Curves exceeding 40° Cobb are likely to progress after growth (Asher & Burton 2006; Goldberg et al. 2002; Kruzel & Moramarco 2020; Lonstein 1995). This fact is often stressed when spine surgeons tell their patients that surgery would be necessary. However, the consequences of this kind of progression are not clarified. Usually, a progression of 0.5–1.0° Cobb per year is calculated. In single thoracic curves, one expects a progression of 0.5° per year, which would add on 10° Cobb within 20 years. In double curves, even less progression is to be expected (Asher & Burton 2006; Goldberg et al. 2002; Kruzel & Moramarco 2020; Lonstein 1995). There is no evidence that this kind of progression will affect the health status of the patient significantly (Asher & Burton 2006; Goldberg et al. 2002; Kruzel & Moramarco 2020; Lonstein 1995; Weinstein et al. 2003; Weiss et al. 2016).

### Health-related quality of life and spinal deformity

There is a significant body of literature about HRQoL in patients with spinal deformities undergoing spinal surgery (Aghdasi et al. 2020). However, the question arises whether from the literature on HRQoL, a surgical indication can be derived? It has already been discussed that studies on HRQoL after surgery are prone to the dissonance effect and may not necessarily reflect all the issues patients might have experienced during or after the operation (Crigger & Meek 2007; Kitayama et al. 2004; Moses, Last & Mahler 1984; Simmons, Webb & Brandon 2004; Stone 2003; Weiss & Goodall 2008b).

### Complications of spine surgery for spinal deformities

Surgical treatment for spinal deformities does not lead to an improvement of health, does not regularly reduce pain and does not necessarily reduce the trunk deformity (Bettany-Saltikov et al. 2016; Cheuk et al. 2015; Hawes 2006; Hawes & O’Brien 2008; Weiss 2008; Weiss & Goodall 2008a, 2009; Ward et al. 2017). Considering the fact that most of the spinal deformities that are treated operatively today such as AIS and Scheuermann’s kyphosis are benign conditions (Turnbull & Weiss 2020; Weinstein et al. 2003; Weiss et al. 2016) and the consequences thereof, usually can be treated conservatively with success (Zapata et al. 2015), no general indication for surgical management can be derived. In view of the long-term unknown considerations and the possible long-term complications of such treatment (Hawes 2006; Moramarco 2013; Mueller & Gluch 2012; Weiss 2007; Weiss & Goodall 2008b; Weiss & Moramarco 2013; Zhang & Zhang 2020), a surgical indication for patients with spinal deformities must be reviewed on an individual basis and considered carefully.

In the short term, spinal fusion surgery today seems a safe procedure; however, the long-term consequences of surgery for spinal surgery are not yet clear (Hawes 2006; Mueller & Gluch 2012; Weiss 2007; Weiss & Goodall 2008b; Zhang & Zhang 2020). In the few studies existing on mid- to long-term complications, the rate of complications is estimated to be 25%–48% of the patients treated surgically (Hawes 2006; Mueller & Gluch 2012; Weiss & Goodall 2008b). Furthermore, some of the long-term complications may not even be attributed to the original surgery (Hawes 2006).

With respect to the lack of evidence for surgery (Bettany-Saltikov et al. 2016; Cheuk et al. 2015; Hawes 2006; Hawes & O’Brien 2008; Ward et al. 2017; Weiss 2008; Weiss & Goodall 2008a, 2009) and the long-term unknown factors (Hawes 2006; Moramarco 2013; Weiss & Goodall 2008b; Weiss et al. 2013) of such procedures, the authors feel that surgery is rarely the most appropriate treatment.

### The appearance of a conflict of interest

The number of long construct spine fusion has increased dramatically since 2004 (Beschloss et al. 2021), for patients aged 65 years and above by as much as 460%. The authors find no explanation for this, and estimate that this increase in long construct spine fusion is because of the improved availability, better safety during surgery and an improved understanding of the spinopelvic parameters.

The question is: Why are AIS patients and patients with a Scheuermann kyphosis of a certain degree of curvature and their parents told that spinal surgery is needed when according to the lack of evidence there obviously is no indication and the risk/reward ratio may be unfavourable for the patient in the long term?

The answer may be a point of view that was not considered by Beschloss et al. (2021): A conflict of interest! (Hawes 2002; Rosen 2020; Weiss 2020). This conflict of interest is addressed in a few scientific articles only (DiPaola et al. 2014; Mirza 2004; Perret & Rosen 2011). Hawes in her book has dedicated a chapter describing the appearance of a conflict of interest (Hawes 2002:107–109).

This conflict of interest has also been discussed in well-known American newspapers and blogs (Abelson 2006; Carreyrou & McGinty 2010; Feder 2006; Oxford University Press 2007; Pollack 2005). In 2005, Medtronic Inc. settled a lawsuit with a spine surgeon who invented a conic screw with the amount of 1.35 billion USD. This was reported in The New York Times (Pollack 2005). The same newspaper also reported on the ‘Spine as a Profit Center’ also addressing the fact that spine surgeons reap many millions of dollars for their ideas for improving spinal implants (Abelson 2006). Also, in other publications, the conflict of interest of spine surgeons has been addressed. In the Wall Street Journal, one can read that in the United States, spine surgeons usually double their income with financial contributions from the implant industry (Carreyrou & McGinty 2010). It is interesting to note that for receiving these contributions, it seems enough to have ‘some ideas’ not necessarily specified. To hold a patent is obviously not a mandatory condition for receiving money from the industry (Rosen 2020).

The Scoliosis Research Society (SRS) holds annual meetings and publishes the conference programme online. All members must disclose their conflict of interest (Scoliosis Research Society 2019:21–48). As can be seen within the programme, nearly all members of the board of directors, most of the committee members, have affiliations with one or more companies involved in the production of spinal implants or related business. Most of these specialists can also be found on the board of the leading journals (e.g. Spine, Journal of Bone and Joint Surgery, Spinal Deformities; see Rosen 2020). It appears that the conflict of interest may therefore affect the scientific literature and – more importantly – the indications for surgery.

There is also some evidence that surgeons may influence national and international healthcare policies in a way to make it easier for the patient to be reimbursed for surgery than for brace treatment (Weiss 2020).

Considering the rising numbers of long spinal fusions (Beschloss et al. 2021) and the obvious lack of evidence only few surgeons seem to tell their patients that with respect to health, there is no huge advantage of surgery when compared to non-surgical management (Ward et al. 2017). Indeed, Farshad et al. (2020) in their article conclude ‘After around 47 and 39 years, respectively, surgical and non-surgical treatment of moderate AIS showed similar subjective outcomes, but with a relevant smaller curve magnitude with surgical treatment’.

This study shows that patients with AIS do not benefit from scoliosis surgery over the long term. The Cobb angle alone was significantly lower in the surgically treated patient group than in the conservatively treated patient group. Despite the differences in the Cobb angle, no differences in the subjectively perceived functional limitations were found in both groups when the Oswestry Disability Index (ODI) was used. Findings like this should be disclosed to the patient in order to support a more objective view on the individual indication for surgery.

Patients need to be informed that many surgeons have lucrative affiliations to one or more implant companies or even own companies themselves (Abelson 2006; Carreyrou & McGinty 2010; Feder 2006; Hawes 2002; Oxford University Press 2007; Pollack 2005; Rosen 2020; Weiss 2020). In order to provide more objective information, the indication for surgery needs to be given by an independent conservative specialist. Otherwise patient’s safety could be compromised by the conflict of interest a large number of surgeons have. In the United States, there is the Association for Ethics in Spine Surgery (2020), where patients can find a spine surgeon without any affiliation to industry. Such associations should also be established in other countries in order to preserve patient’s safety.

### Limitations

This article contains a narrative review of the literature. With a review extended to more databases on the basis of a systematic review, more contributions may be found. However, as earlier and more recent systematic reviews did not find any high-quality evidence based on prospective controlled or randomised studies, it does not seem reasonable to assume that a high-quality article would have been overlooked. In order to support the conclusions drawn in this article and to elaborate a more objective ‘risk reward ratio’ for the individual patient, a current systematic review with meta-analysis is indicated.

## Conclusions

Surgical treatment for spinal deformities according to our narrative review does not lead to an improvement of health, does not always reduce pain and does not necessarily reduce the trunk deformity. Therefore, a general indication for spine surgery just based on the Cobb angle is not given.

In view of the long-term unknown variables and the possible long-term complications of such treatment, a surgical indication for patients with spinal deformities must be reviewed on an individual basis and considered carefully.

Any conflict of interest a spine surgeon has must be disclosed to the patient and/or legal guardian when advising for a surgical intervention.

A current systematic review appears necessary in order to be able to draw final conclusions on the indication for surgery in patients with spinal deformities.
